# The relationship between gut microbiota and insomnia: a bi-directional two-sample Mendelian randomization research

**DOI:** 10.3389/fcimb.2023.1296417

**Published:** 2023-11-28

**Authors:** Yan Li, Qingqing Deng, Zhanli Liu

**Affiliations:** Department of Neurology, Hangzhou Children’s Hospital, Hangzhou, Zhejiang, China

**Keywords:** gut microbiome, insomnia, sleep disorders, bi-directional Mendelian randomization analysis, relationship

## Abstract

**Introduction:**

Insomnia is the second most common mental health issue, also is a social and financial burden. Insomnia affects the balance between sleep, the immune system, and the central nervous system, which may raise the risk of different systemic disorders. The gut microbiota, referred to as the “second genome,” has the ability to control host homeostasis. It has been discovered that disruption of the gut-brain axis is linked to insomnia.

**Methods:**

In this study, we conducted MR analysis between large-scale GWAS data of GMs and insomnia to uncover potential associations.

**Results:**

Ten GM taxa were detected to have causal associations with insomnia. Among them, class *Negativicutes*, genus *Clostridiuminnocuumgroup*, genus *Dorea*, genus *Lachnoclostridium*, genus *Prevotella7*, and order *Selenomonadalesare* were linked to a higher risk of insomnia. In reverse MR analysis, we discovered a causal link between insomnia and six other GM taxa.

**Conclusion:**

It suggested that the relationship between insomnia and intestinal flora was convoluted. Our findings may offer beneficial biomarkers for disease development and prospective candidate treatment targets for insomnia.

## Introduction

Insomnia disorder, defined by self-reported sleep difficulties, is characterized by persistent difficulty initiating or sustaining sleep as well as related daytime dysfunction. With 10% to 20% of the population affected, insomnia is the second most common mental health issue (after anxiety disorder), and it is more common in older people and women. In adults, 5.8% to 20% of the population suffers from insomnia, but the prevalence of insomnia in the elderly ranges from 30% to 48%. Insomnia disorder is among the top 10 reasons for general practitioners’ consultations (Lo Yun et al., 2022). It is also a social and financial burden, raising questions about public health. Insomnia affects the balance between sleep, the immune system, and the central nervous system, which may raise the risk of infection, depression, cardiovascular disease, gastrointestinal disorders, and respiratory illnesses. Chronic insomnia contributes to a variety of negative outcomes, including decreased physical and mental health (e.g., cardiovascular disease and stroke), worsened health-related life quality, and poorer mental health (e.g., chronic pain, anxiety, depression, substance misuse, and suicide). Given the severity of the negative impacts of insomnia, identifying risk factors is essential for treatments (Jia et al., 2022; Yao et al., 2022; Gibson et al., 2023).

The intestinal flora, also referred to as the “second genome,” has the ability to control host homeostasis, which includes metabolic rate, immune/inflammatory response, and cardiovascular function (Le Chatelier et al., 2013). The gut microbiome (GM) is also linked to neuropsychiatric illnesses, as it may regulate brain function and behavior through the microbiota-gut-brain axis (Iannone et al., 2019; Zhang et al., 2021; Wang Z. et al., 2022). There are variations in GM taxa among people with epilepsy, depression, autistic spectrum disorder, and Parkinson’s disease. Recent research has shown that the gut-brain axis is dysregulated in relation to insomnia and that abnormalities in the gut microbiota can make the condition worse. To date, there have been few investigations into the relationship between intestinal flora and insomnia (Qi et al., 2022; Bundgaard-Nielsen et al., 2023; Chalet et al., 2023).

Fortunately, large-scale genome-wide association studies (GWASs) on gut microbiota and insomnia are now available, allowing for a meaningful assessment of association in MR analysis. Through instrumental variables (IV) that are genetic variants strongly related to the exposure of interest, Mendelian randomization (MR) analysis is used to investigate the causal relationship between exposure and outcome. In MR research, single nucleotide polymorphisms (SNPs) are used as instrumental variables (IV) (Burgess and Thompson, 2017; Burgess et al., 2017). SNPs adhere to the principle of random genetic variation assignment at meiosis, which eliminates the influence of confounding factors and the potential impact of reverse causation because genetic variants exist prior to the start of the disease (Lawlor et al., 2008). Therefore, when compared to RCT, MR analysis can more quickly identify the causal relationships between relevant exposure components and outcomes. Currently, no MR studies on insomnia and GM have been undertaken. Here, we conduct an MR analysis through large-scale GWAS summary statistics of GMs and insomnia to uncover potential GM taxa that could support some current findings and offer novel viewpoints on the identification and management of insomnia.

## Materials and methods

### Study design

**Figure 1:**
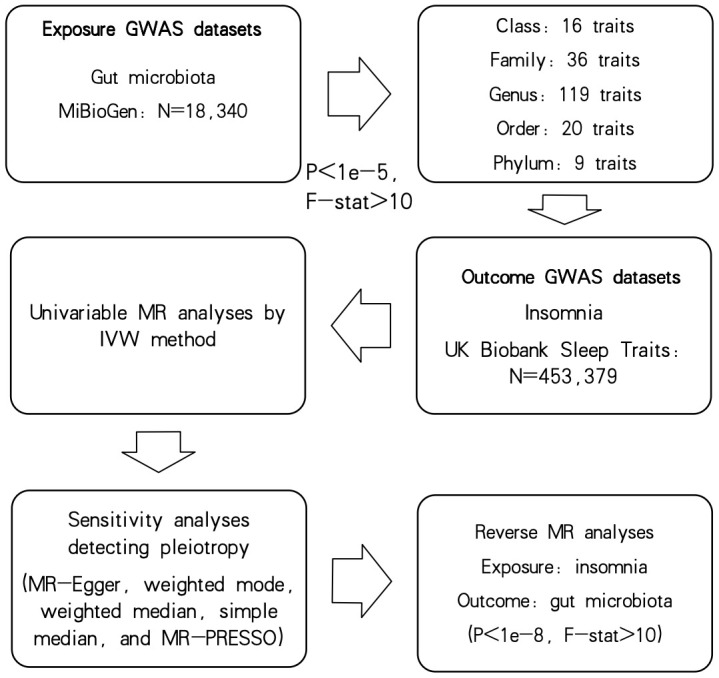
Flowchart of current study.

The overall flow chart of this study is shown in **Figure 1**. The three presumptions below must be satisfied by MR studies: (i) IVs are highly linked with exposure variables, (ii) IVs are independent of confounding factors, and (iii) IVs are only associated with outcomes via exposure factors (Burgess et al., 2017). Our results followed the STROBE-MR guidelines (Skrivankova et al., 2021a).

### Data sources of the exposure and outcome

From the MiBioGen project, we obtained the gut microbiota statistics summary-level data, the largest genome-wide meta-analysis to date (Kurilshikov et al., 2021). In the MiBioGen project, the 16S rRNA gene sequencing profiles of 18,340 individuals were assembled and evaluated, and 211 GM taxa were identified (from genus to phylum level, including 9 phyla, 16 classes, 20 orders, 35 families, and 131 genera). The GWAS summary statistics for GMs can be found at https://mibiogen.gcc.rug.nl (Swertz and Jansen, 2007; Swertz et al., 2010; van der Velde et al., 2019). Insomnia GWAS summary data were obtained from the UK Biobank Sleep Traits GWAS: Self-report (insomnia associations and sleep duration associations) (https://sleep.hugeamp.org/downloads.html). Insomnia symptoms were self-reported by European-ancestry UK Biobank participants (n=453,379). Participants were asked the question: “Do you have trouble falling asleep at night or do you wake up in the middle of the night?” and were asked to select from responses of “never/rarely”, “sometimes”, “usually”, and “prefer not to answer” in this sample; twenty-nine percent of people self-reported experiencing frequent insomnia symptoms on a regular basis (or “usually”) (Gibson et al., 2023).

### Identification of IVs

In this MR study, IVs were SNPs that were highly correlated with each GM taxon. We obtained the number of IVs for gut microbiota data with the threshold (p<5×10^-5^), and the threshold in reverse MR analyses was set under p<5×10^-8^ for insomnia data. Additionally, we removed SNPs within a window size of 500 kb and a threshold of r^2^<0.1 to reduce linkage disequilibrium (LD) for gut microbiota data, whereas for insomnia data in reverse MR analysis, the window size was set at kb=10000 and a threshold of r^2^<0.001. Then, we eliminated palindromic SNPs and SNPs that were not present in the IV results. Finally, to measure the degree of weak instrumental bias, the F-statistic of IVs was computed. If the F-statistic was >10, it was assumed that no bias was caused by weak IVs. The formula for calculating the F-value is $F=\left(\frac{R^{2}}{1-R^{2}}\right)\frac{\left(n-k-1\right)}{k}$ , $R^{2}=2\times (1-MAF)\times MAF\times {\left(\beta \right)}^{2}$ (Pierce et al., 2011).

### Statistical methods

The principal MR method for determining causation was the inverse variance weighted random effect (IVW-RE). Based on the meta-analysis principles, the IVW approach is a Wald ratio estimator extension (Pagoni et al., 2019). Methods of MR-Egger, weighted median, simple mode, and weighted mode were carried out for each GM taxon on insomnia (Bowden et al., 2016; Burgess and Thompson, 2017; Davies et al., 2018). If the IVW approach revealed a causal association for that taxon (p<0.05), these four MR methods were used to supplement the IVW findings. The criterion of the weighted median method is that at least 50% of the SNPs must satisfy the premise that they are valid IVs (Davies et al., 2018; Verbanck et al., 2018). The MR-Egger method provides unbiased estimates even when all selected IVs are multivariate (Burgess and Thompson, 2017). Finally, odds ratios (OR) and 95% confidence intervals (CI) were utilized to present the findings of causal connections. The significance cutoff was established at p<0.05.

Only exposure-outcome pairs that were discovered using all MR techniques and had the same direction were thought to have a causal relationship. We also carried out a number of sensitivity studies to examine the consistency of the causal association. First, horizontal pleiotropy was identified through the MR-Egger and MR-PRESSO tests (Rees et al., 2017; Verbanck et al., 2018; Skrivankova et al., 2021b). Additionally, the leave-one-out and Funnel plots analyses were conducted to evaluate the reliability of the findings. In this study, “TwoSampleMR” and “MR-PRESSO” packages of R software (version 4.3.0) were used to carry out the MR analysis. 

To investigate the reverse causality of insomnia (as exposures) on gut microbiota (as outcomes), a reverse MR analysis was carried out for insomnia on each GM taxa. The process followed the same guidelines as the methodology indicated above for the two-sample MR. This bidirectional MR and sensitivity analysis follows the rules of the TwoSample MR and MR-PRESSO packages.

## Results

### Two-sample Mendelian randomization of gut microbiota (exposure) on insomnia (outcome)

#### Details of IVs

Under a suggestive significance level of P<1×10^-5^, 2,284 SNPs were discovered, and three duplicated SNPs (rs10805326, rs2728491, and rs2704155) were deleted. These SNPs were grouped into five categories as final IVs: class, family, order, genus, and phylum. Particularly, there were 200 IVs in 18 classes, 439 IVs in 35 families, 245 IVs in 20 orders, 1,483 IVs in 131 genera, and 112 IVs in 9 phyla. Furthermore, all IVs were shown to be more strongly related to exposure than to outcome (*p*_exposure_<*p*_outcome_), and all F-statistics were greater than 10. Details of the IVs of insomnia are presented in **Supplementary Table 1**.

### MR analysis

**Figure 2:**
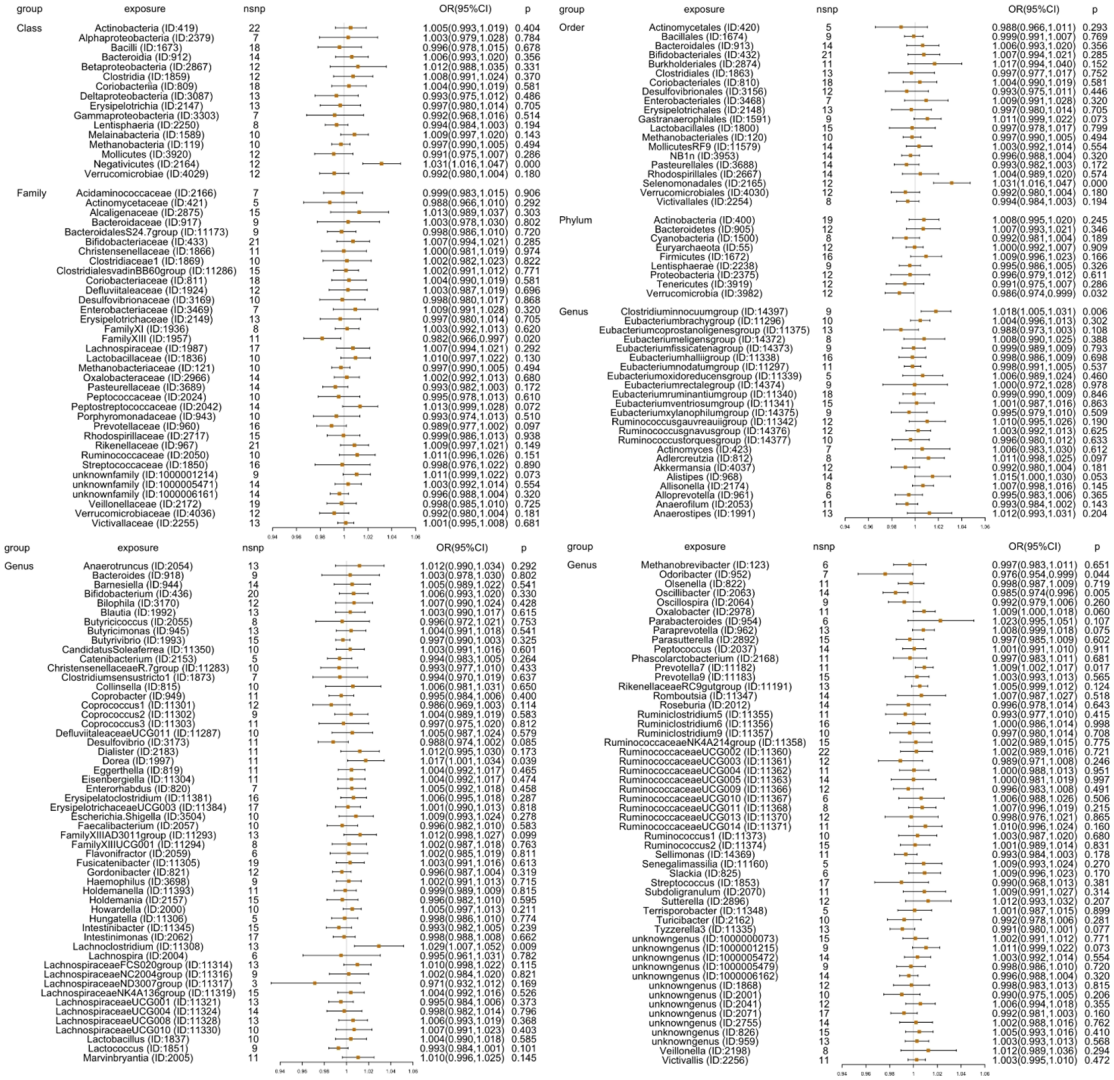
Results [OR (95%CI)] for MR analyses for GWAS data of 211 GM taxa (exposure) on insomnia (outcome) through inverse variance weighted random effect (IVW-RE) method: 9 phyla, 16 classes, 20 orders, 35 families, and 131 genera. It showed that 10 GM taxa, including class *Negativicutes* (ID: 2164), Family *FamilyXIII* (ID: 1957), order *Selenomonadales* (ID: 2165), phylum *Verrucomicrobia* (ID: 3982), genus *Odoribacter* (ID: 952), genus *Oscillibacter* (ID: 2063), genus *Clostridiuminnocuumgroup* (ID: 14397), genus *Dorea* (ID: 1997), genus *Lachnoclostridium* (ID: 11308), and genus *Prevotella7* (ID: 11182), had causality with insomnia.

First, 211 GM taxa with five methods (IVW-RE, MR-Egger, weighted median, simple mode, and weighted mode) were evaluated using MR analysis to determine their causal relationship with insomnia (**Figure 2**). The IVW-FE results revealed that 10 GM taxa had a significant association with insomnia. Family *FamilyXIII* (ID: 1957) [OR=0.982 (0.966, 0.997), *p*=0.020], genus *Odoribacter* (ID: 952) [OR=0.976(0.954,0.999), *p*=0.044], genus *Oscillibacter* (ID: 2063) [OR=0.985(0.974,0.996), *p*=0.005], and phylum *Verrucomicrobia* (ID: 3982) [OR=0.986(0.974,0.999), *p*=0.032] were related to a lower risk for insomnia, while class *Negativicutes* (ID: 2164) [OR=1.031(1.016,1.047), *p*=7.53E-05], genus *Clostridiuminnocuumgroup* (ID: 14397) [OR=1.018(1.005,1.031), *p*=0.006], genus *Dorea* (ID: 1997) [OR=1.017(1.001,1.034), *p*=0.039], genus *Lachnoclostridium* (ID: 11308) [OR=1.029(1.007,1.052), *p*=0.009], genus *Prevotella7* (ID: 11182) [OR=1.009(1.002,1.017), *p*=0.017], and order *Selenomonadales* (ID: 2165) [OR=1.031(1.016,1.047), *p*=7.53E-05] were associated with a higher risk of insomnia. Additionally, the findings of Cochran’s Q test showed that there was no heterogeneity.

**Figure 3:**
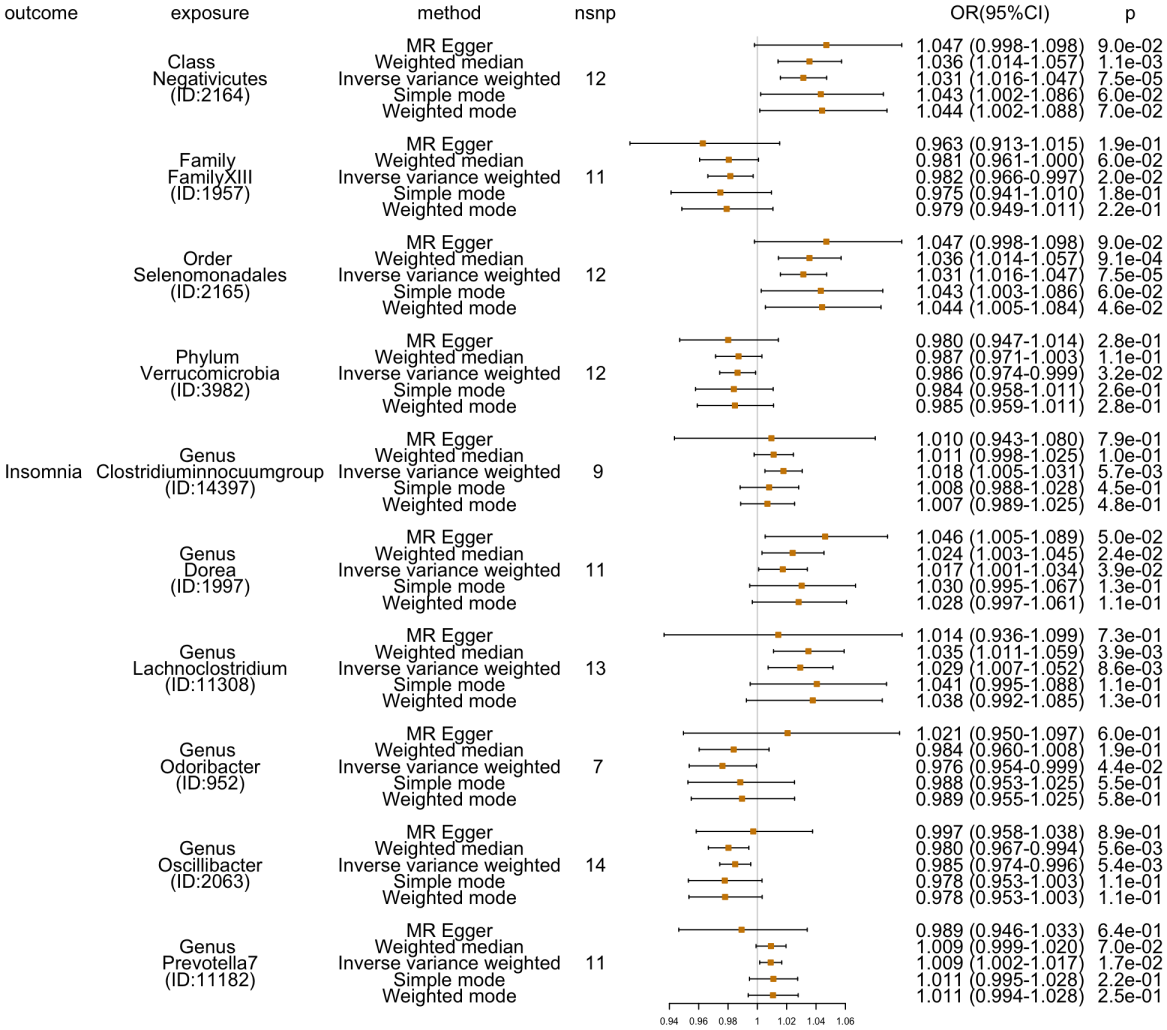
Five methods’ (MR-Egger, weighted median, inverse variance weighted, simple mode, and weighted mode) results (OR[95%CI]) of MR analyses for 10 GM taxa on insomnia.

Additionally, four additional methods, MR-Egger, weighted median, simple mode, and weighted mode, were performed to assess the causal effect of these 10 GM taxa on insomnia (**Figure 3**). The results were consistent with the IVW-FE results. Family *FamilyXIII* (ID: 1957), phylum *Verrucomicrobia* (ID: 3982), genus *Odoribacter* (ID: 952), and genus *Oscillibacter* (ID: 2063) were related with a lower risk for insomnia, while the other six GMs [class *Negativicutes* (ID: 2164), genus *Clostridiuminnocuumgroup* (ID: 14397), genus *Dorea* (ID: 1997), genus *Lachnoclostridium* (ID: 11308), genus *Prevotella7* (ID: 11182), and order *Selenomonadales* (ID: 2165)] showed a higher risk of insomnia. There was no indication of heterogeneity, pleiotropy, or weak instrument bias in the heterogeneity (IVW test and MR-Egger regression), pleiotropy (MR-PRESSO test and MR-Egger regression), or weak instrument bias (F statistic) tests. Additional details are summarized in **Supplementary Table 2**.

### Reverse Mendelian randomization analysis of insomnia (exposure) on gut microbiota (outcome)

IVs were retrieved from GWAS datasets of insomnia in a previous MR analysis of the relationship between gut microbiota and insomnia, with a significance of P-value at 1×10^-8^. Forty SNPs were discovered from 2,500 SNPs (SNPs of insomnia GWAS data at P<1×10^-8^) after removing SNPs with linkage disequilibrium. First, we conducted MR analysis to determine the relationship between insomnia and 10 GM taxa, including the class *Negativicutes* (ID: 2164), family *FamilyXIII* (ID: 1957), genera *Odoribacter* (ID: 952), *Oscillibacter* (ID: 2063), *Clostridiuminnocuumgroup* (ID: 14397), *Dorea* (ID: 1997), *Lachnoclostridium* (ID: 11308), *Prevotella7* (ID: 11182), order *Selenomonadales* (ID: 2165), and phylum *Verrucomicrobia* (ID: 3982). It demonstrated the lack of a causal relationship between insomnia and these 10 GM taxa, which was consistent with our prior MR findings. Additional details are summarized in **Supplementary Table 3** and **Supplementary Figure S1**.

**Figure 4:**
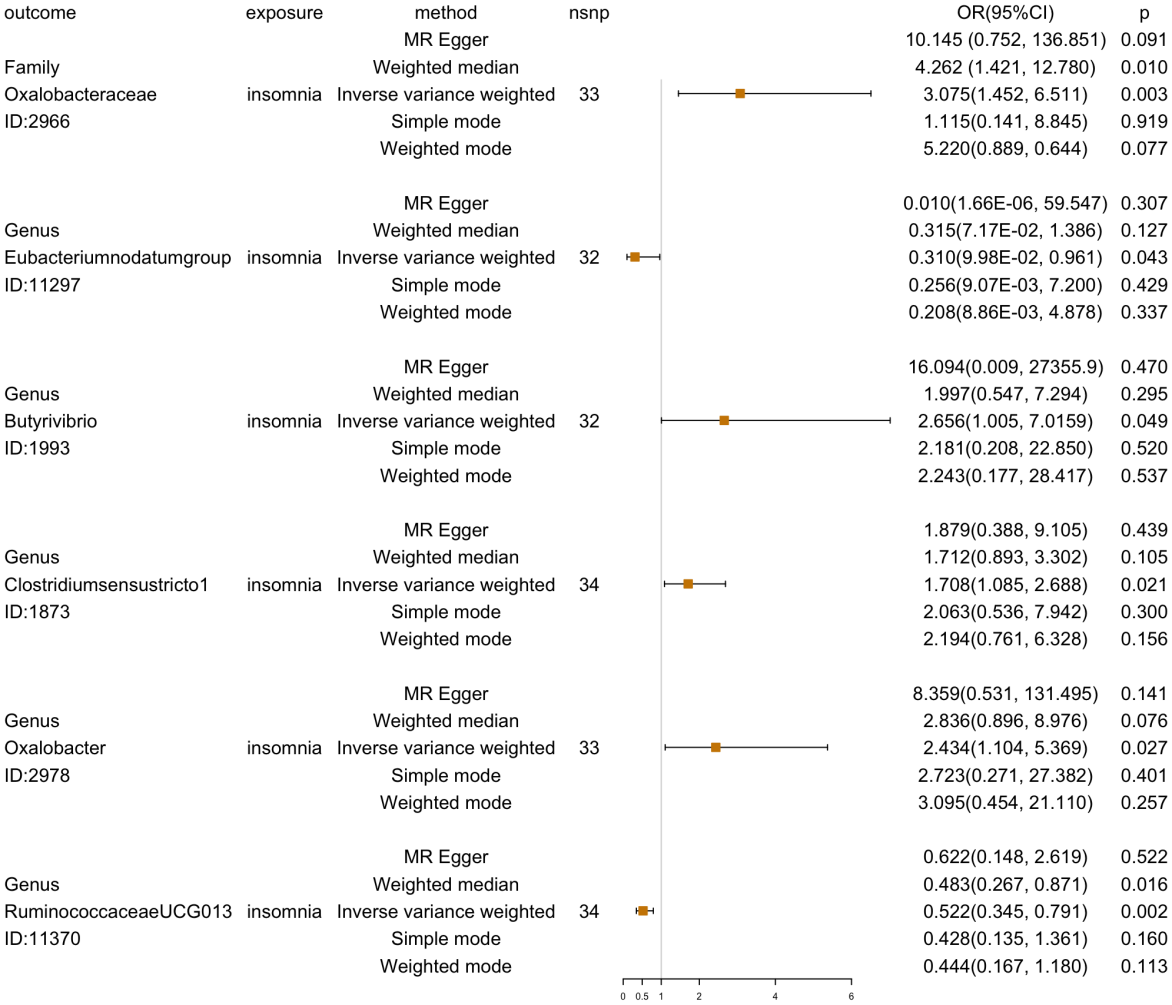
Reverse MR analyses results (OR[95%CI]) of five methods (MR-Egger, weighted median, inverse variance weighted, simple mode, and weighted mode) for insomnia on six GM taxa (family *Oxalobacteraceae*, genus *Butyrivibrio*, genus *Clostridiumsensustricto1*, genus *Oxalobacter*, genus *Eubacteriumnodatumgroup*, and genus *RuminococcaceaeUCG013*).

**Figure 5:**
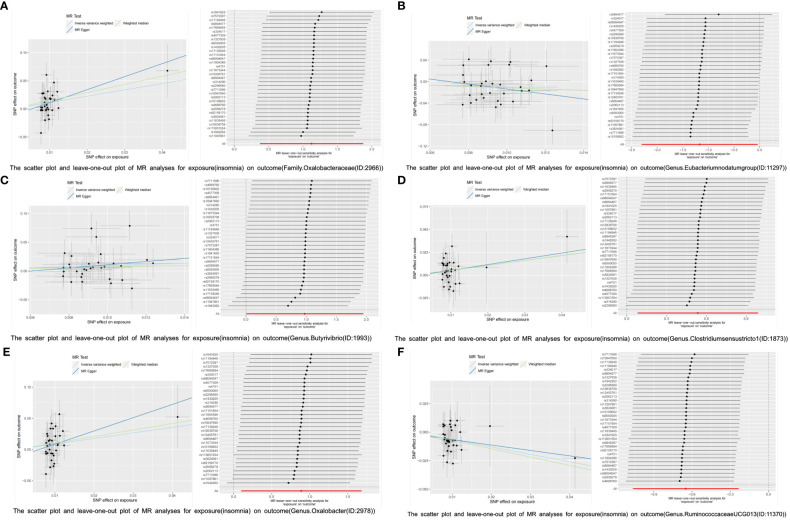
The scatter plot and leave-one-out plot of MR analyses for insomnia on six GM taxa. **(A)**: exposure: insomnia, outcome: family *Oxalobacteraceae* (ID:2966). **(B)**: exposure: insomnia, outcome: genus *Eubacteriumnodatumgroup* (ID:11297). **(C)**: exposure: insomnia, outcome: genus *Butyrivibrio* (ID:1993). **(D)**: exposure: insomnia, outcome: genus *Clostridiumsensustricto1* (ID:1873). **(E)**: exposure: insomnia, outcome: genus *Oxalobacter* (ID:2978). **(F)**: exposure: insomnia, outcome: genus *RuminococcaceaeUCG013* (ID:11370).

Reverse MR analysis was then used to investigate the other 201 GM taxa for insomnia. According to the IVW-FE results, six GM taxa substantially correlate with insomnia. Insomnia could increase the abundance of the gut microbiota of family *Oxalobacteraceae* (ID:2966) [OR=3.075 (1.453, 6.511), *p*=0.003], genus *Butyrivibrio* (ID:1993) [OR=2.656(1.005, 7.016), *p*=0.049], genus *Clostridiumsensustricto1* (ID:1873) [OR=1.708 (1.085, 2.687), *p*=0.021], and genus *Oxalobacter* (ID:2978) [OR=2.434(1.104,5.370), *p*=0.028], while insomnia could decrease the abundance of genus *Eubacteriumnodatumgroup* (ID:11297) [OR=0.310(0.098,0.961), *p*=0.042] and genus *RuminococcaceaeUCG013* (ID:11370)[OR=0.522(0.345, 0.791), *p*=0.002]. Furthermore, four additional methods, MR-Egger, weighted median, simple mode, and weighted mode, were performed to assess the causal effect of insomnia on these GM taxa (**Figure 4**). The outcomes matched those of the IVW in a similar way. In this investigation, neither the IVW test nor the MR-Egger regression showed any evidence of heterogeneity, pleiotropy, or weak instrument bias. The MR-PRESSO test and the MR-Egger regression also showed no evidence of these phenomena. Then, we conducted scatter plots and leave-one-out plots for insomnia on six GM taxa [family *Oxalobacteraceae* (ID:2966), genus *Butyrivibrio* (ID:1993), genus *Clostridiumsensustricto1* (ID:1873), genus *Oxalobacter* (ID:2978), genus *Eubacteriumnodatumgroup* (ID:11297), and genus *RuminococcaceaeUCG013* (ID:11370)]. Furthermore, the inverse variance weighted, MR-Egger, and weighted median results of the MR Steiger directionality test demonstrated a strong direction from insomnia to the six GM taxa. The robustness of our findings was demonstrated by the leave-one-out sensitivity analysis, which showed that no one SNP drives a causal association (**Figure 5**). Funnel plots of Inverse variance weighted and MR Egger results excluded a potential bias (**Supplementary Figure S2**).

## Discussion

To the best of our knowledge, this is the first MR investigation using huge GWAS summary-level data to indicate a probable causal connection between gut microbiota and insomnia. This study examined the causative impact of 211 GM taxa (from the class, family, order, genus, and phylum level) on insomnia. In this study design, we checked and confirmed the assumptions of Mendelian Randomization (MR). We discovered 10 GM taxa that are connected to insomnia, and reverse MR analysis revealed that 6 GM taxa may be affected. The gut microbiome’s potential protective or contributing effects on insomnia suggest a close connection between the two conditions.

Nowadays, the gut microbiome and its impact on humans are receiving increasing attention. Growing data suggest that the gut microbiota (GM) can control host homeostasis in both health and disease, for example, through the gut-brain axis, gut-lung axis, gut-kidney axis, gut-skin axis, gut-liver axis, and gut-immune axis. Researchers have discovered links between gut microbiota and many diseases, such as autism spectrum disorder, depression, epilepsy, Alzheimer’s disease, type 2 diabetes, obesity, chronic obstructive pulmonary disease, atopic dermatitis, COVID-19 illness, psoriasis, and systemic autoimmune diseases. Clinical trials on intestinal flora have shown efficacy in the treatment of disorders such as epilepsy, autism, Alzheimer’s disease, inflammatory bowel disease, rheumatoid arthritis, and psoriasis (Iannone et al., 2019; Orru et al., 2020; Mahmud et al., 2022; Mousa et al., 2022; Qin et al., 2022). Recent research has shown that GMs are involved in neuropsychiatric disorders because they affect brain activity and behavior through the microbiota-gut-brain axis (Johnson and Foster, 2018). It has been discovered that disruption of the gut-brain axis is linked to insomnia. In our study, we identified six GMs in the reverse MR. Given that gut microbiota was related to many diseases, these disorders may directly or indirectly arise in the six GMs. The gut microbiota’s particular metabolites have been reported to be related to insomnia, and alterations in the gut microbiota may worsen the condition. Its molecular mechanism is not yet fully understood (Haimov et al., 2022; Feng et al., 2023).

Insomnia is believed to have a negative impact on the quality of life in adults and the elderly population around the world. In general, insomnia affects 5.8 to 20% of the adult population, whereas it affects 30% to 48% of the elderly population. Insomnia is the result of a complex interaction of behavioral (such as stress, lifestyle, workplace culture, environment, and sleeping arrangements), physiological, and genetic factors. The negative effects of insomnia on many organs lead to abnormal sleep patterns, cognitive performance, and emotional reactions (Li et al., 2020; Jia et al., 2022; Gibson et al., 2023). In addition to contributing to the pathological progression of the immunological, endocrine, and cardiovascular systems, it also causes neuropsychiatric illnesses such as depression, dementia, mania, schizophrenia, and anxiety disorders. The risk of hypertension, diabetes mellitus, arthritis, stomach ulcers, gastroesophageal reflux illness, migraine, depression, obesity, heart attack or stroke, asthma, menstruation issues, obesity, and infection has also been linked to insomnia. These conditions and consequences have a cumulative effect on insomnia. Multiple attempts have been made to build models to interpret and explain the onset and evolution of insomnia; nevertheless, these models are insufficient to represent comprehensive knowledge (Reynolds et al., 2017; Gao et al., 2019; Cai et al., 2021; Jiang et al., 2022; Wang Q. et al., 2022; Zeng et al., 2023).

In this study, through MR analysis of the GWAS database, we investigated whether there is a connection between intestinal flora and insomnia. The findings demonstrated a strong relationship and potential interaction between gut flora and insomnia. Studies have suggested that gut bacteria play a role in the development of insomnia, but the specific mechanism is unknown. Growing evidence points to a critical function for the gut microbiota in the regulation of sleep behavior, both directly and indirectly, as well as a potential role in the pathophysiology and etiology of sleep disorders. It has been found that in older people with insomnia, differences in the composition of the gut microbiota and the abundance of particular genera are associated with poor sleep and poor cognitive function. Studies revealed that insomniacs had considerably higher relative abundances of *Lactobacillus crispatus* and *Streptococcus* compared to healthy controls. Five metabolic pathways, including those for glycerophospholipid metabolism, glutathione metabolism, nitrogen metabolism, aspartate, glutamate, alanine metabolism, and aminoacyl-tRNA production, may be involved in the gut microbiota’s ability to cause insomnia (Le Chatelier et al., 2013; Ning et al., 2022). In both human studies and animal models, it has been suggested by researchers that gut bacteria may contribute to sleep issues.

When compared to the findings of earlier studies, this study’s findings exhibit parallels and discrepancies. Pro-inflammatory activation may be one major component causing insomnia. According to studies, chronic sleep deprivation is linked to higher levels of IL-1 and TNF-α in the brain, as well as higher levels of IL-6 in the blood during the day. According to research (Wang Q. et al., 2022), people with acute and chronic insomnia disorders have lower abundances of several anaerobic gut flora taxa, including *Lachnospira*, *Roseburia*, and *Prevotella 9*. We discovered that the genera *Prevotella7* (ID:11182) and *Lachnoclostridium* (ID:11308) are associated with a significant incidence of insomnia. *Prevotella* is a Gram-negative bacterium that helps break down protein and carbohydrate foods. *Prevotella* is frequently believed to have a lower abundance in certain diseases (Ley, 2016). Studies (Feng et al., 2023) also identified that several *Prevotella* (such as *Prevotella amnii*, *Prevotella buccalis*, *Prevotella colorans*, and *Prevotella timonensis*) were associated with changes in inflammatory and metabolite levels, indicating that *Prevotella* may affect sleep by regulating metabolites and promoting inflammation. However, more is not always better; recent human research has showed an increase in *Prevotella* to systemic illnesses such as periodontitis, bacterial vaginosis, rheumatoid arthritis, metabolic problems, low-grade systemic inflammation, and schizophrenia (Bertelsen et al., 2021; Iljazovic et al., 2021). The gut taxon *Lachnoclostridium* is a genus of Gram-positive bacteria, and people with ulcerative colitis and irritable bowel syndrome tend to have higher concentrations of *Lachnoclostridium* (Cai et al., 2022). *Lachnoclostridium* was more prevalent in patients with COVID-19 and was identified by MR analysis as having a high risk of AD (Iannone et al., 2019; Zhang and Zhou, 2023).

In contrast to earlier research, our findings show that the genus *Dorea* (ID:1997) has a high risk of sleeplessness. According to studies (Zhang et al., 2021), patients with major depressive illnesses and sleep disorders had lower levels of *Streptococcus*, *Dorea*, *Barnesiella*, and *Intestinibacter*. *Dorea* bacteria is a member of the thick-walled bacterial porophyllium group, which is widely distributed in the human intestine. By inducing Treg cells and preventing Th17 cell differentiation and function, *Dorea* bacteria can control the intestinal immune response and preserve the stability and integrity of the gut mucous barrier. *Dorea* is more prevalent and is suspected to have an inflammatory effect in patients with multiple sclerosis, inflammatory bowel disease, colorectal cancer, autism spectrum disorders, and obesity. Studies have shown that the composition, diversity, and metabolic activity of the gut microbiota change significantly between healthy individuals and insomniacs. *Bacteroides* and *Clostridiales* are considered to be the two most crucial biomarkers for differentiating between insomniacs and healthy people. Additionally, the mechanism behind the connection between gut microbiota and insomnia is unclear. Research has found that the microbial ecosystem in the human gut is complex and diverse, and the collaborative relationships between different types of bacteria can disrupt the stability of the microbial ecosystem; the competitive relationship between different bacterial communities helps maintain the stability of the intestinal ecosystem. Rakoff-Nahoum S (Rakoff-Nahoum et al., 2016) evolved cooperation within the *Bacteroidales*, the dominant Gram-negative bacteria of the human intestine. We know little about cooperation within this important ecosystem and studies are few. Our research provides guidance and a foundation for the management of insomnia. This current study is also incomplete, we did not identify GMs and the associated SNPs to understand the meaning of the SNPs to the GM. It will be very meaningful to figure out the function of SNPs to the GMs in the future.

The current study has a number of limitations that need to be mentioned. Firstly, because the study only included participants with primarily European ancestry, there could be already many genomic variations within European ancestry. It may be possible that the SNPs in the host can directly or indirectly cause insomnia. Therefore, in order to strengthen the conclusion, additional research involving participants from diverse parts of the world would be necessary to extend the findings to other groups without constraint. Secondly, a higher permissive threshold (p<1×10^-5^) was used because there were so few IVs that met the rigorous criteria (p<5×10^-8^) for screening. Thirdly, self-reporting insomnia symptoms has limitations, including recall bias and lack of granularity; the cases of insomnia in this study were not strictly defined, so future analysis based on strict criteria for insomnia GWAS data is required to strengthen confidence in a conclusion. Fourthly, the GM GWAS data included in this analysis was based solely on 16S rRNA sequencing from genus to phylum level; additional metagenomic and multiomic techniques should target gut microbiota composition at a more precise level to prevent bias. Fifthly, MR analysis relies on three important assumptions mentioned above. In this study, SNPs are used as instrumental variables (IV), GMs as exposure, and insomnia as outcome (GMs>SNPs>insomnia); in reverse MR analysis, insomnia is used as exposure and GMs as outcome (insomnia>SNPs>GMs); considering the complicated links between insomnia and GMs, it may be possible that host SNPs may affect insomnia through GMs; another MR study (GMs as IV, SNPs as exposure, and insomnia as outcome; SNPs>GMs>insomnia) could be conducted to explore the causality between host SNPs and insomnia in the future. Finally, the identified GMs might exist in both insomnia and healthy individuals; therefore, it is necessary to regroup the individuals with or without the GM-associated significant IVs, and intervening with GMs in insomnia research can help strengthen the causal link in the future.

## Conclusion

Overall, we detected 10 causal associations after performing an MR analysis on the causal impacts of 211 GM taxa on insomnia. Among them, class *Negativicutes*, genus *Clostridiuminnocuumgroup*, genus *Dorea*, genus *Lachnoclostridium*, genus *Prevotella7*, and order *Selenomonadalesare* were significantly associated with increased insomnia risk. We discovered a causal link between insomnia and six other GM taxa through reverse MR analysis. It suggested that the relationship between insomnia and intestinal flora is convoluted. However, since the current work was based on a GWAS summary-level dataset derived from 16S rRNA sequencing, more in-depth analyses based on more advanced large-scale studies generated from metagenomics sequencing are required. Nevertheless, our findings may offer beneficial biomarkers for disease development and prospective candidate treatment targets for insomnia.

## Data availability statement

The datasets presented in this study can be found in online repositories. The names of the repository/repositories and accession number(s) can be found in the article/**Supplementary Material**.

## Ethics statement

This study of “The relationship between gut microbiota and insomnia: a bi-directional two-sample Mendelian randomization research” relied on publicly available de-identified data from participant studies that had been authorized by an ethical standards committee. There was hence no need for extra, separate ethical approval for this investigation. Written informed consent for participation was not required from the participants or the participants’ legal guardians/next of kin in accordance with the national legislation and institutional requirements.

## Author contributions

YL: Conceptualization, Data curation, Formal Analysis, Funding acquisition, Investigation, Methodology, Project administration, Software, Visualization, Writing – original draft, Writing – review & editing. QD: Data curation, Supervision, Writing – review & editing. ZL: Project administration, Supervision, Visualization, Writing – review & editing.
