# Supplementary figures and images for: The relationship between gut microbiota and insomnia: a bi-directional two-sample Mendelian randomization research
Source: Front Cell Infect Microbiol. 2023 Nov 28;13:1296417. doi: 10.3389/fcimb.2023.1296417 (PMC10714008; doi:10.3389/fcimb.2023.1296417)

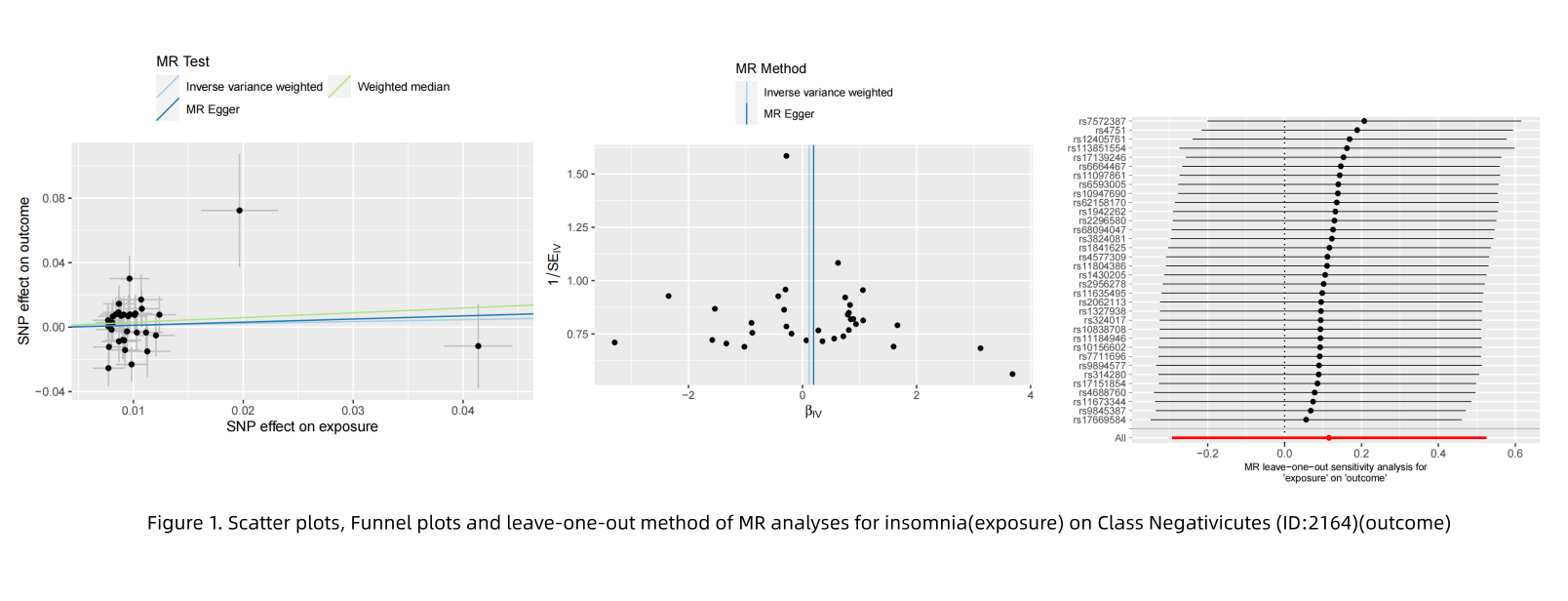


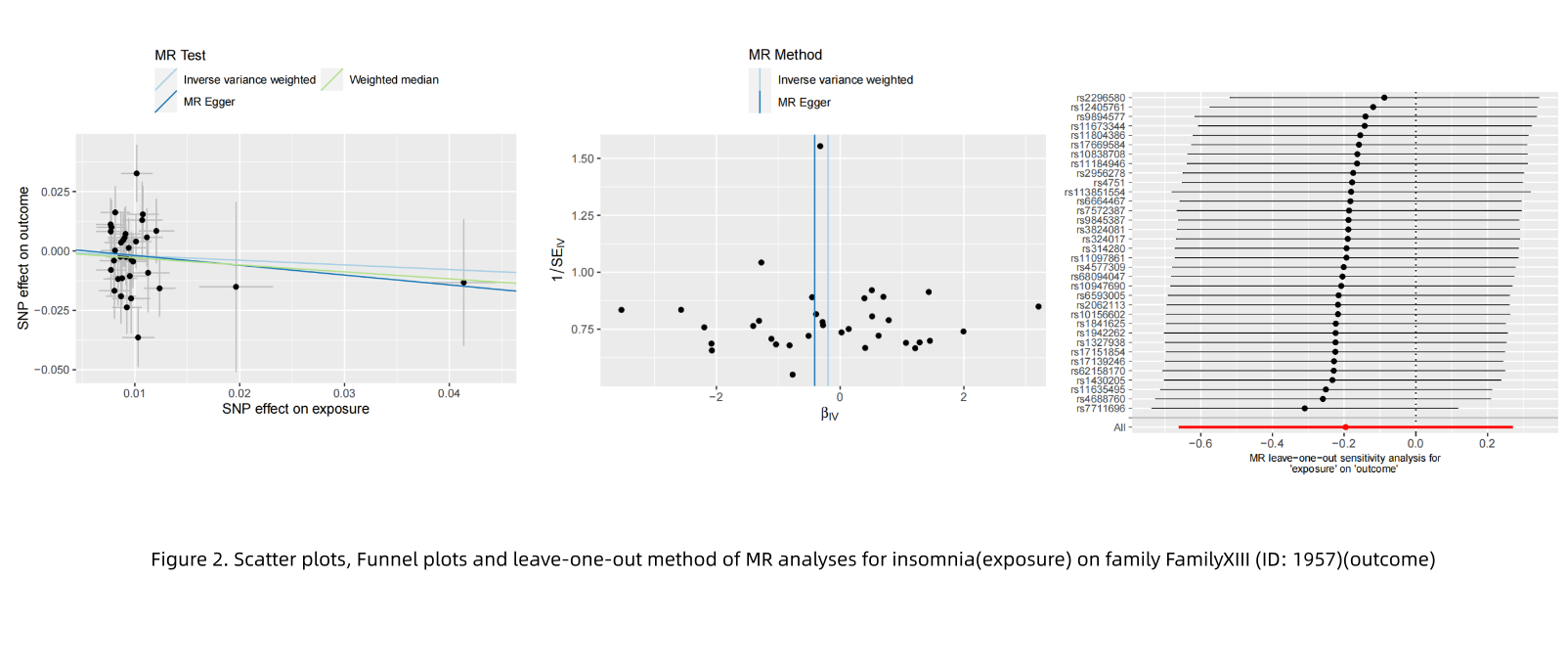


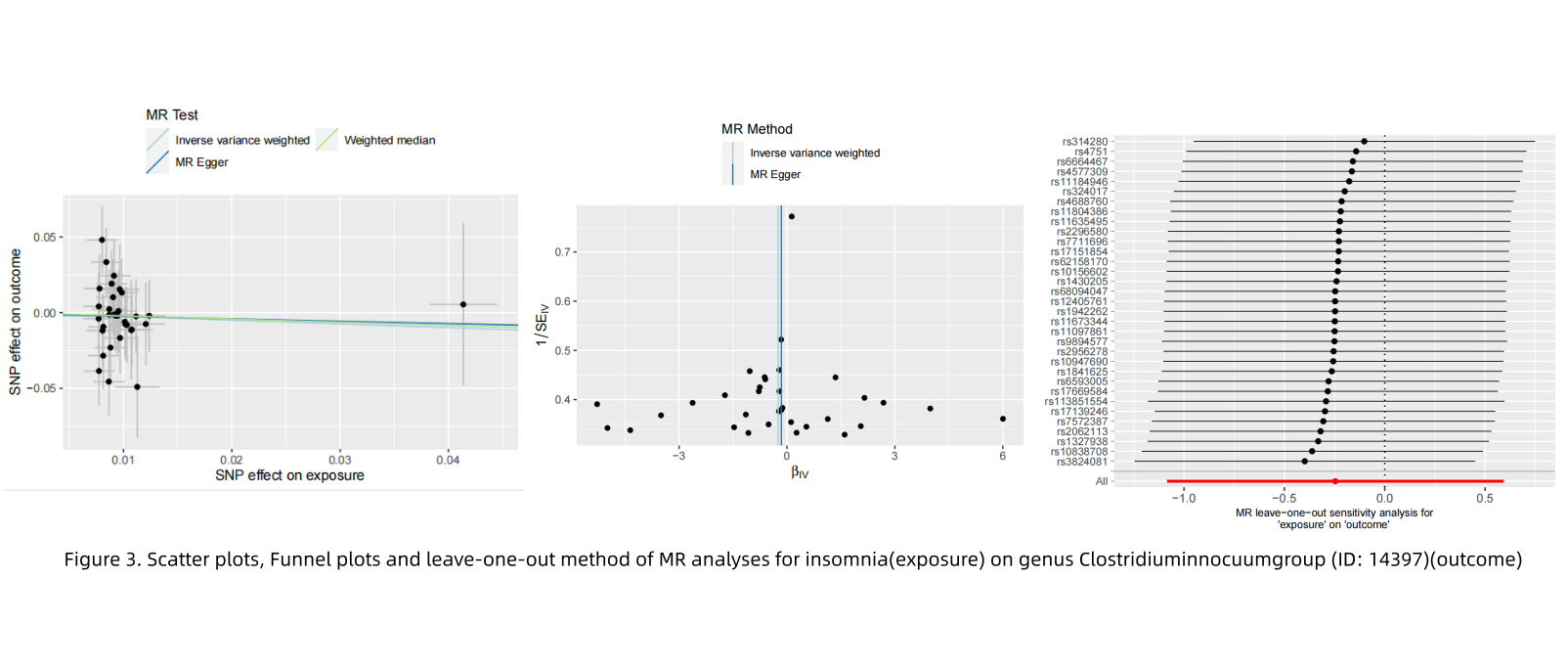


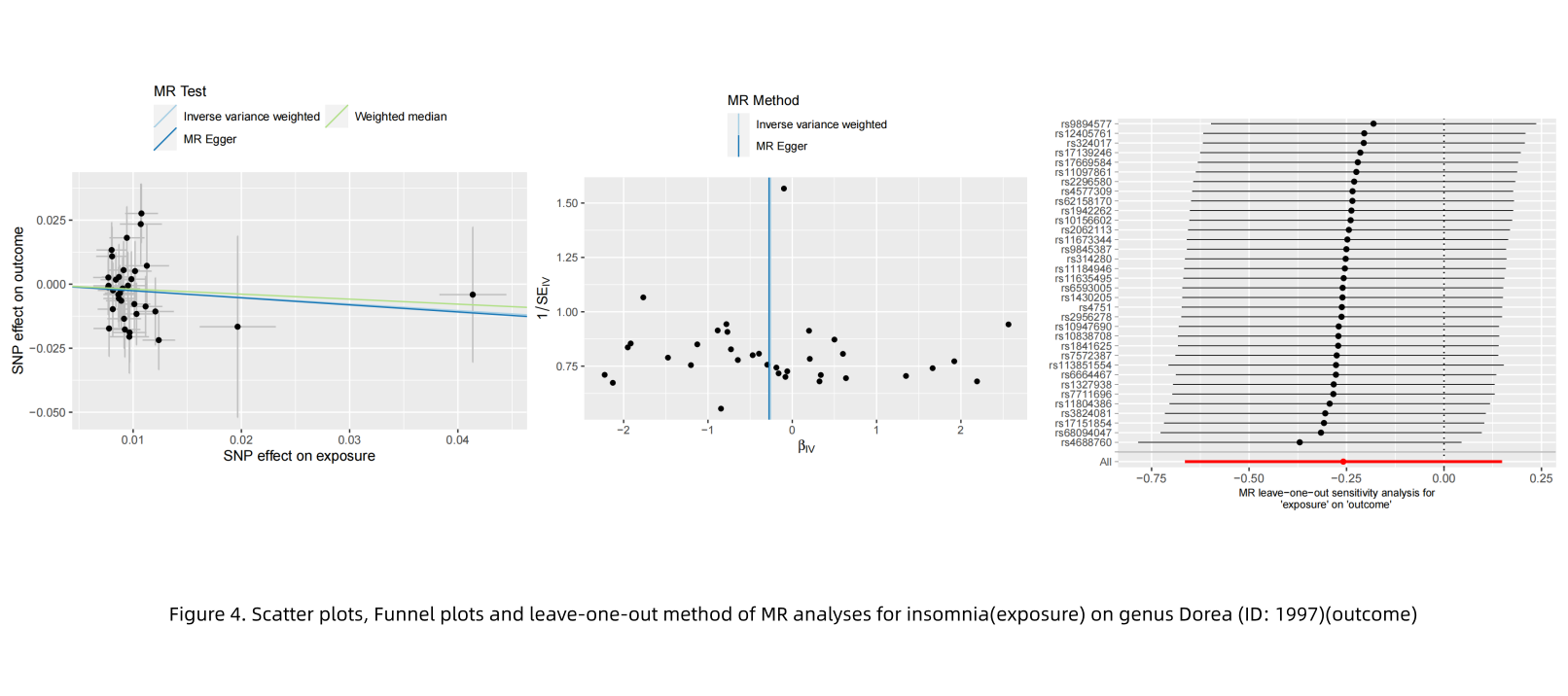


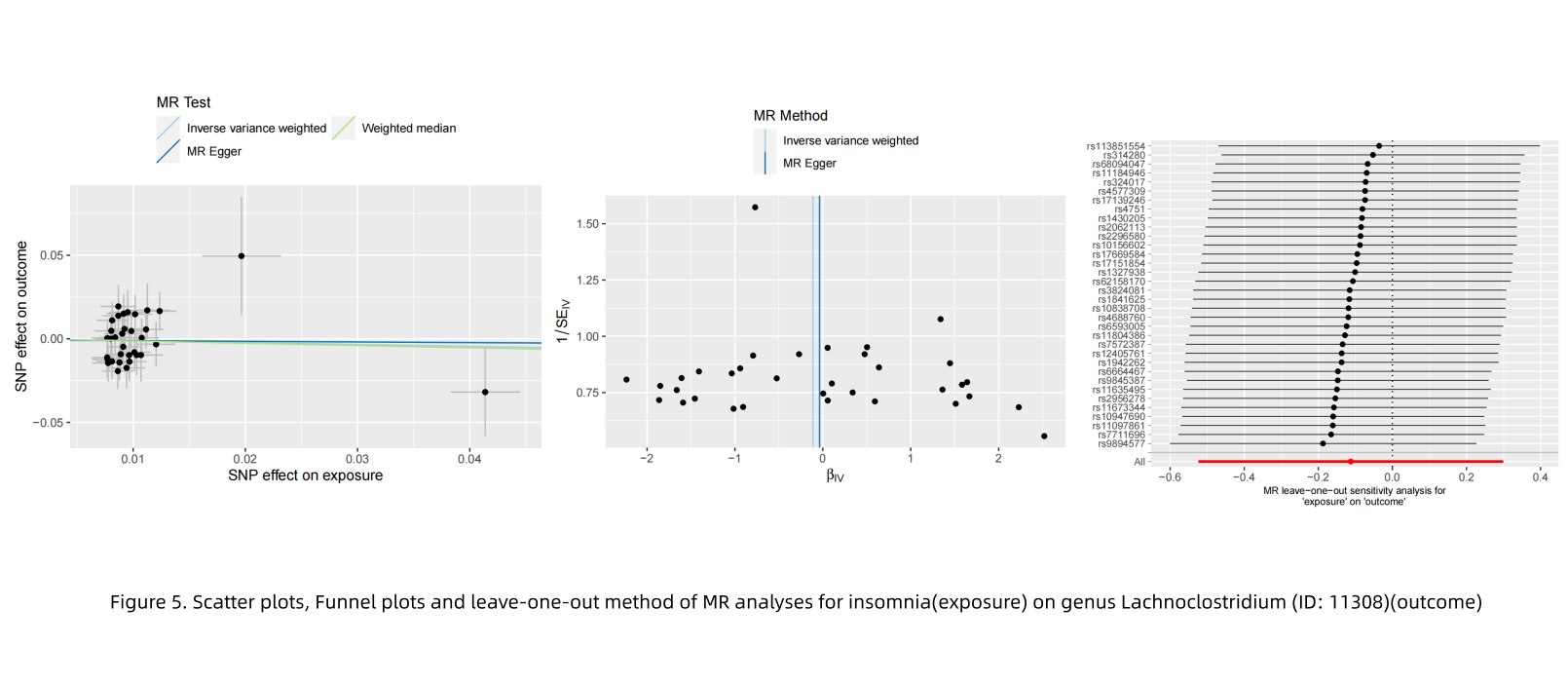


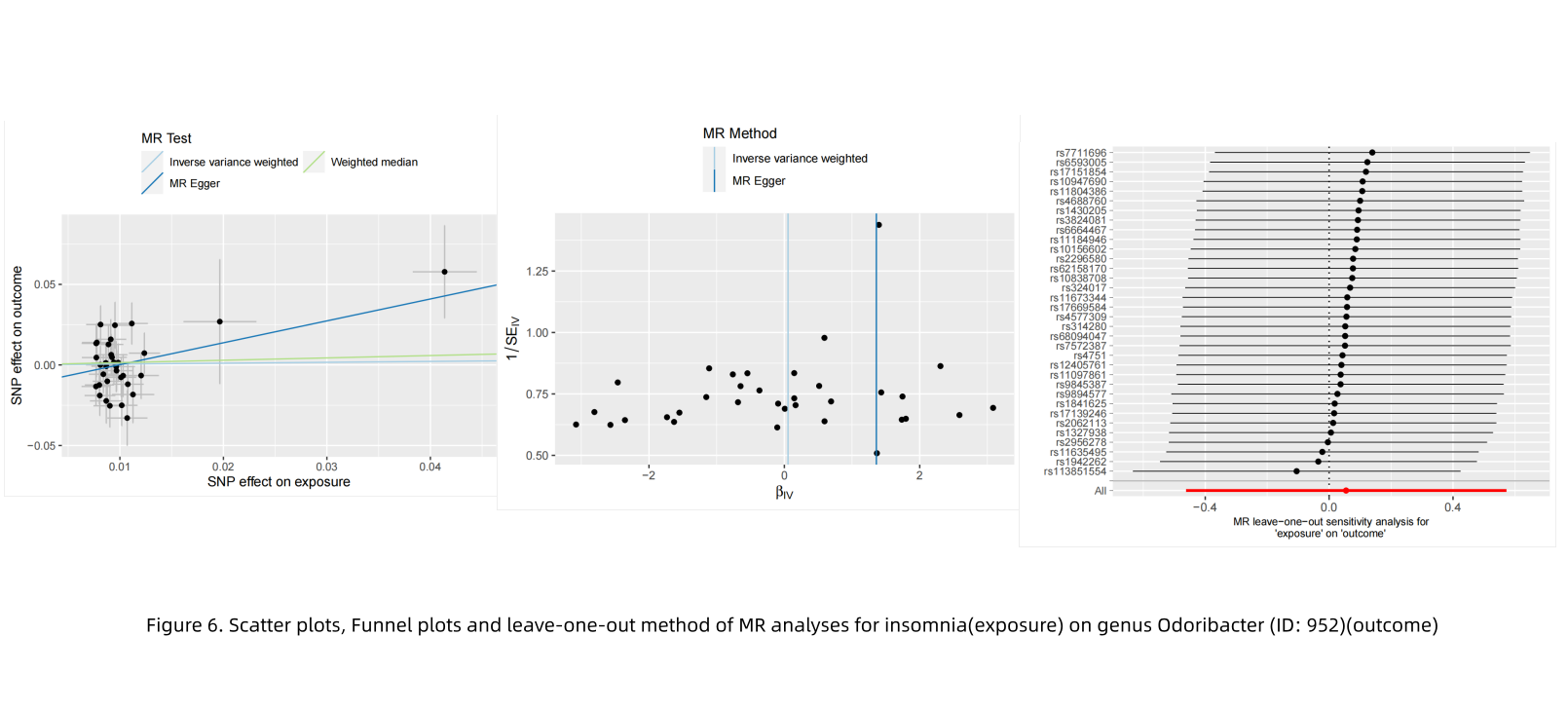


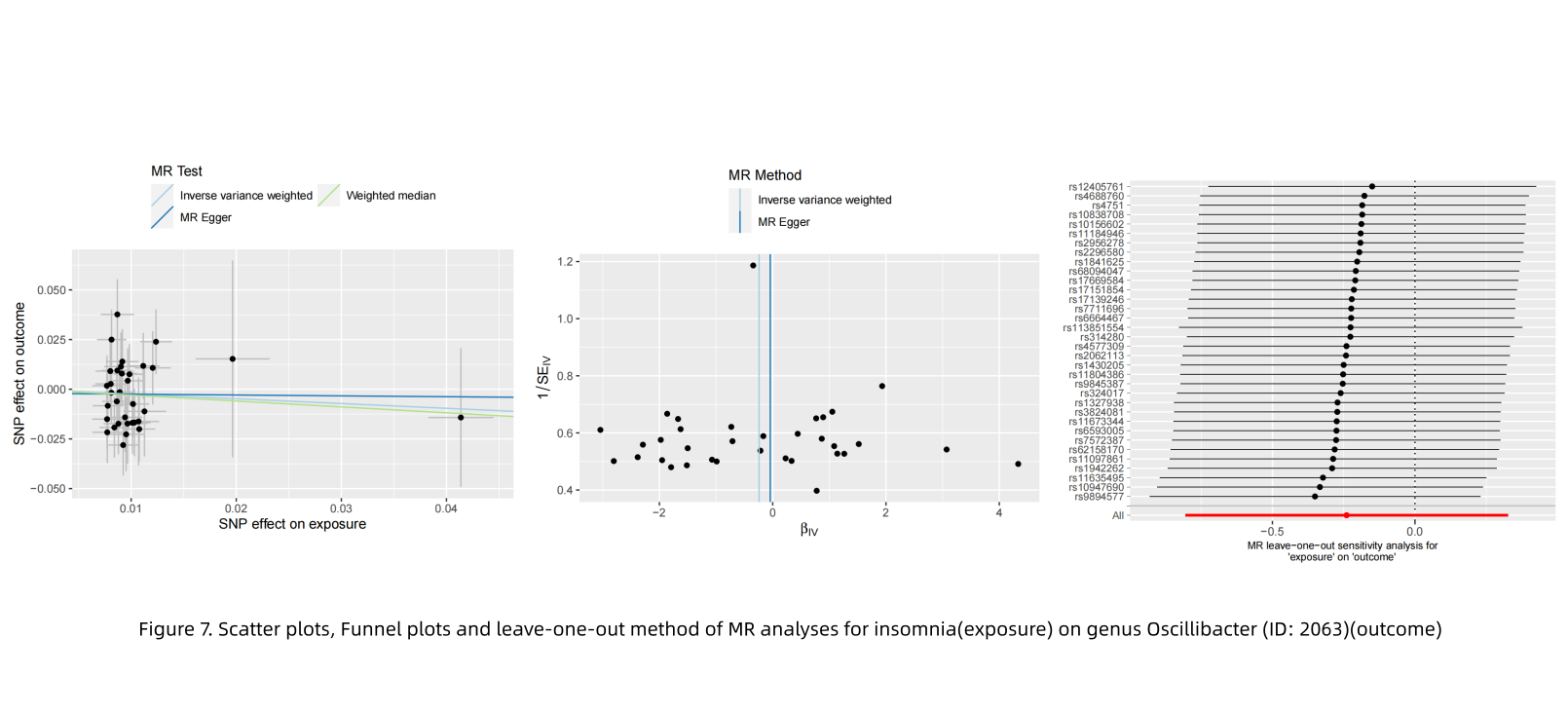


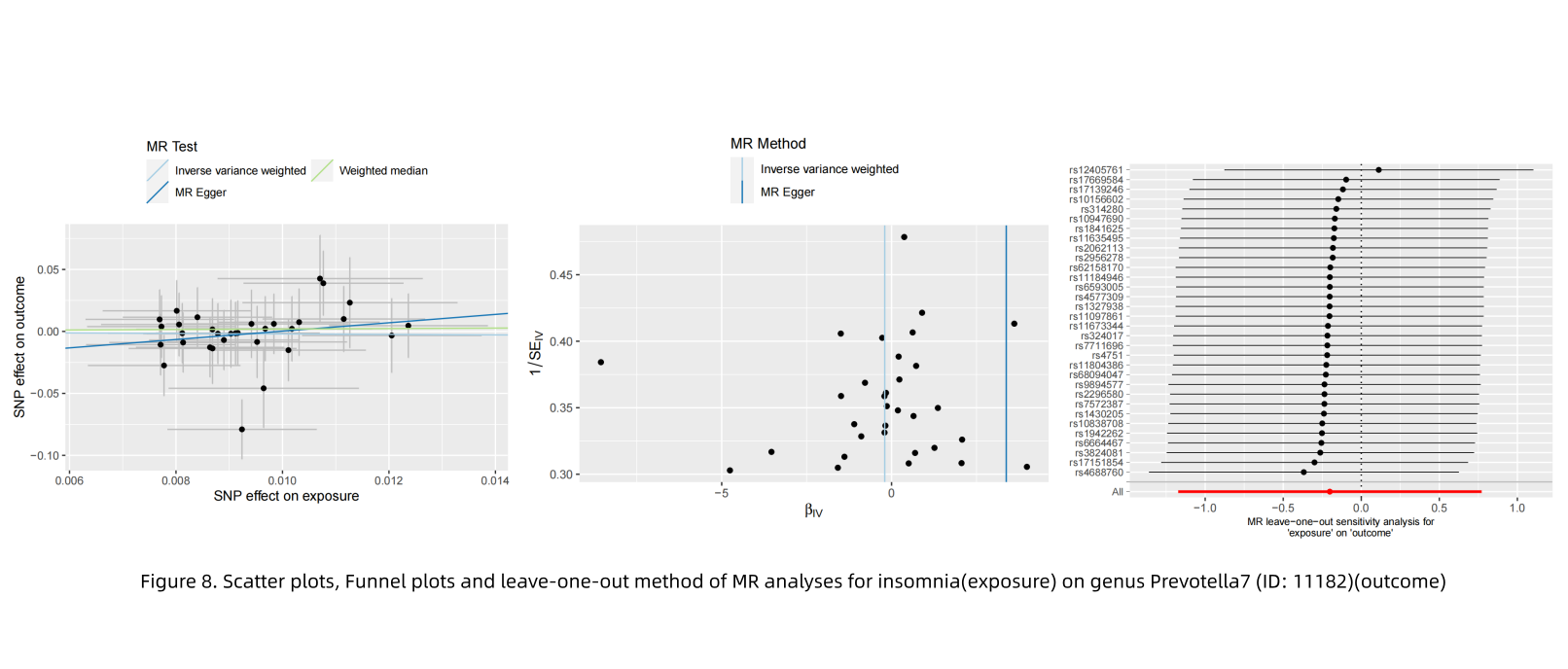


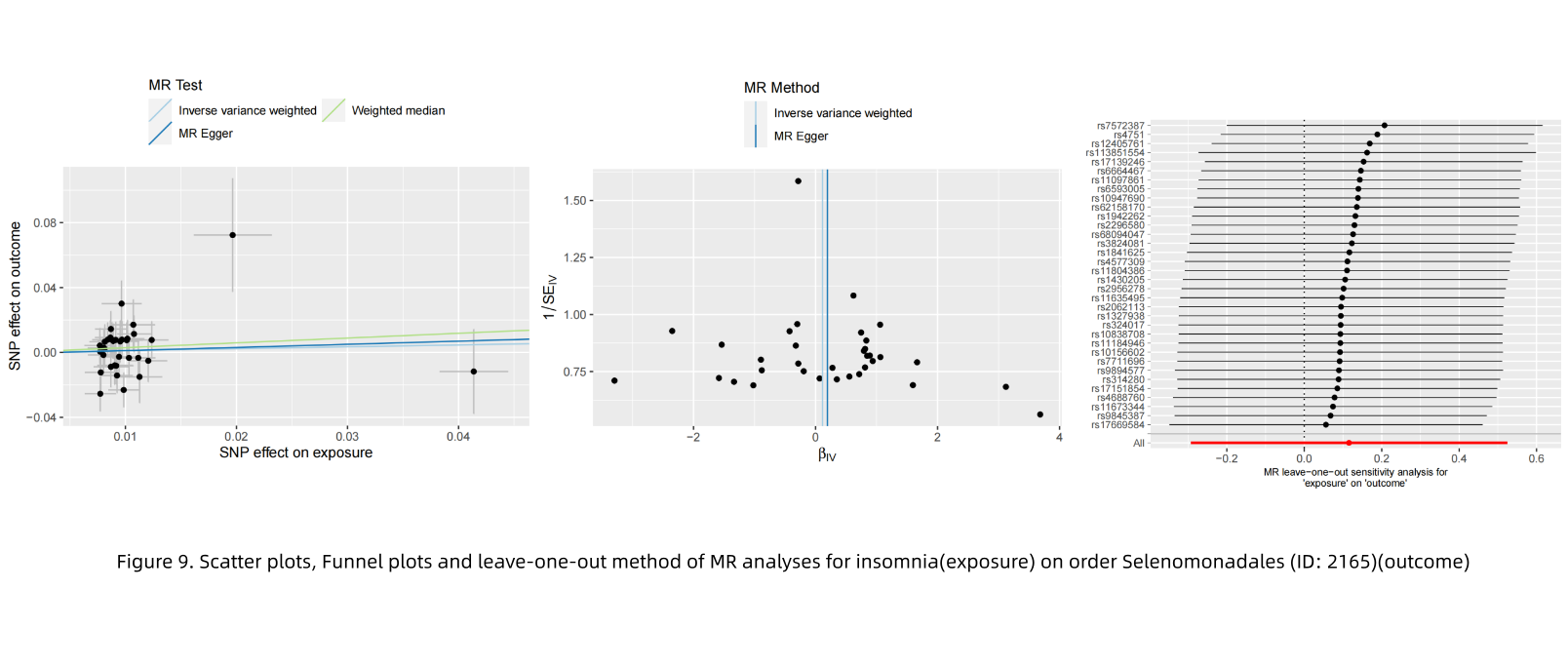


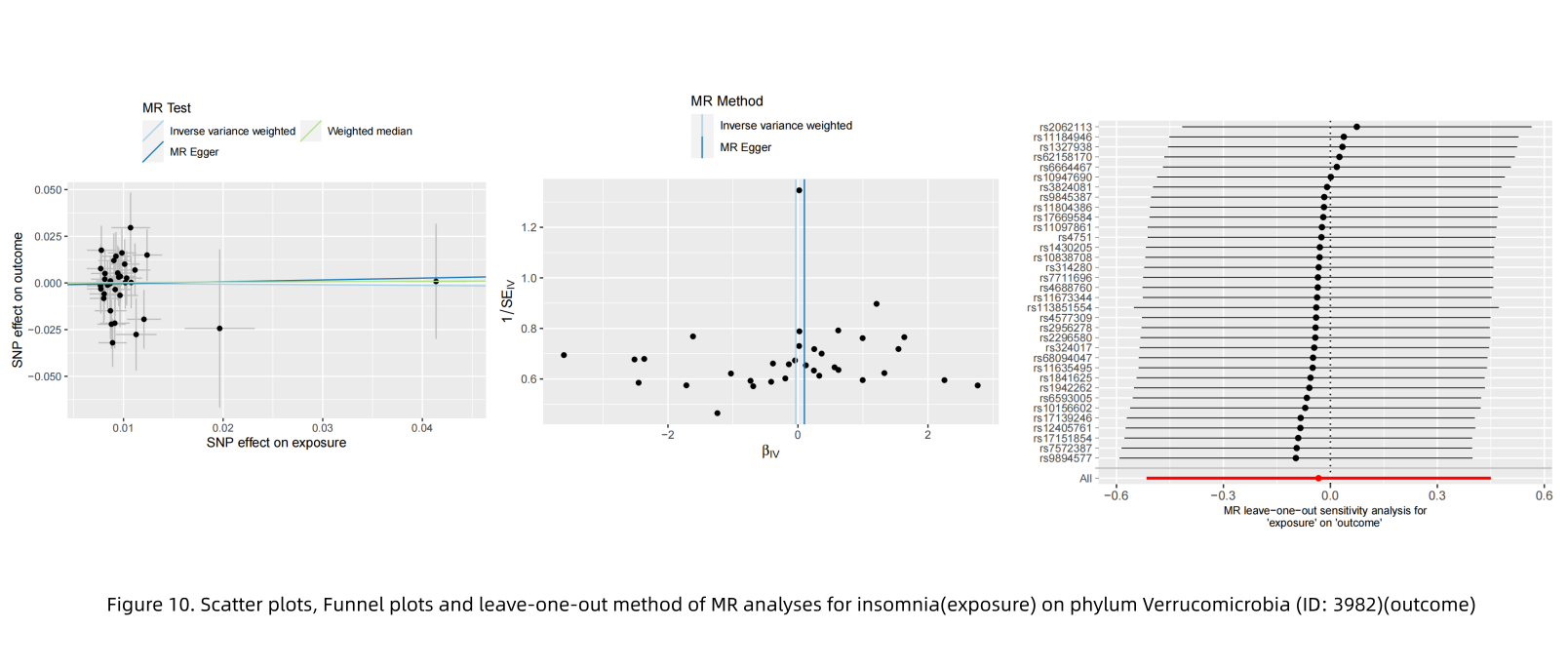

Supplement: Supplementary file 1 [file DataSheet_1.docx]
