# Supplementary material for: The relationship between gut microbiota and insomnia: a bi-directional two-sample Mendelian randomization research
Source: Front Cell Infect Microbiol. 2023 Nov 28;13:1296417. doi: 10.3389/fcimb.2023.1296417 (PMC10714008; doi:10.3389/fcimb.2023.1296417)
Supplement: Supplementary file 2 [file DataSheet_2.docx]

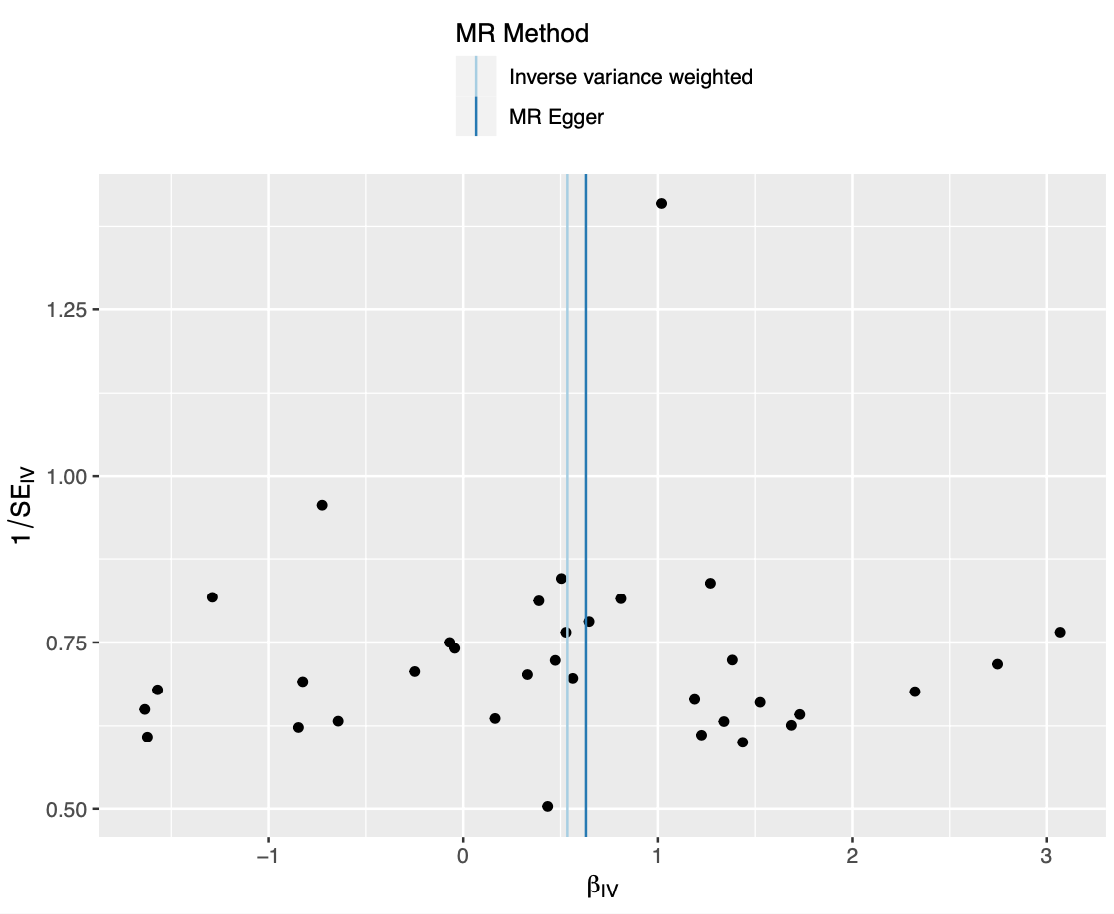


Figure 1. Funnel plots of MR analyses for insomnia on genus *Clostridiumsensustricto1* (ID:1873)


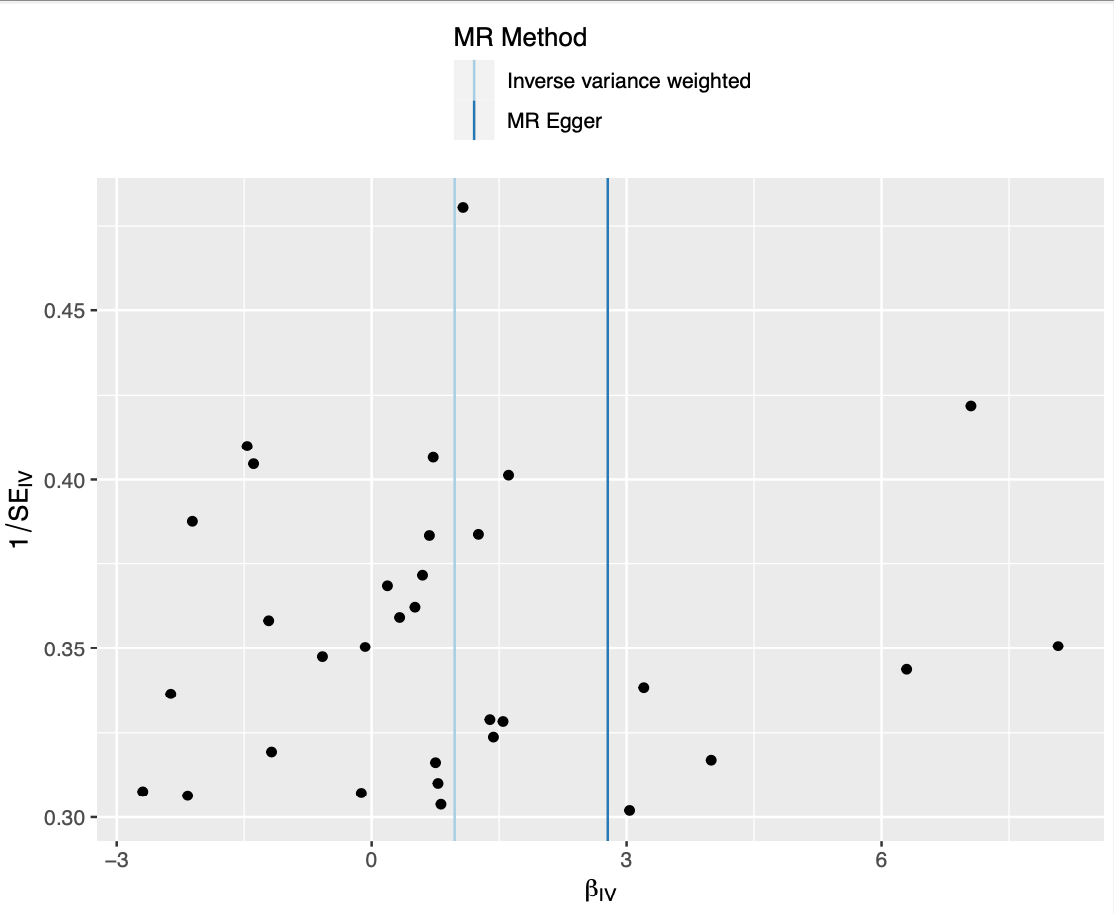


Figure 2. Funnel plots of MR analyses for insomnia on genus *Butyrivibrio* (ID:1993)


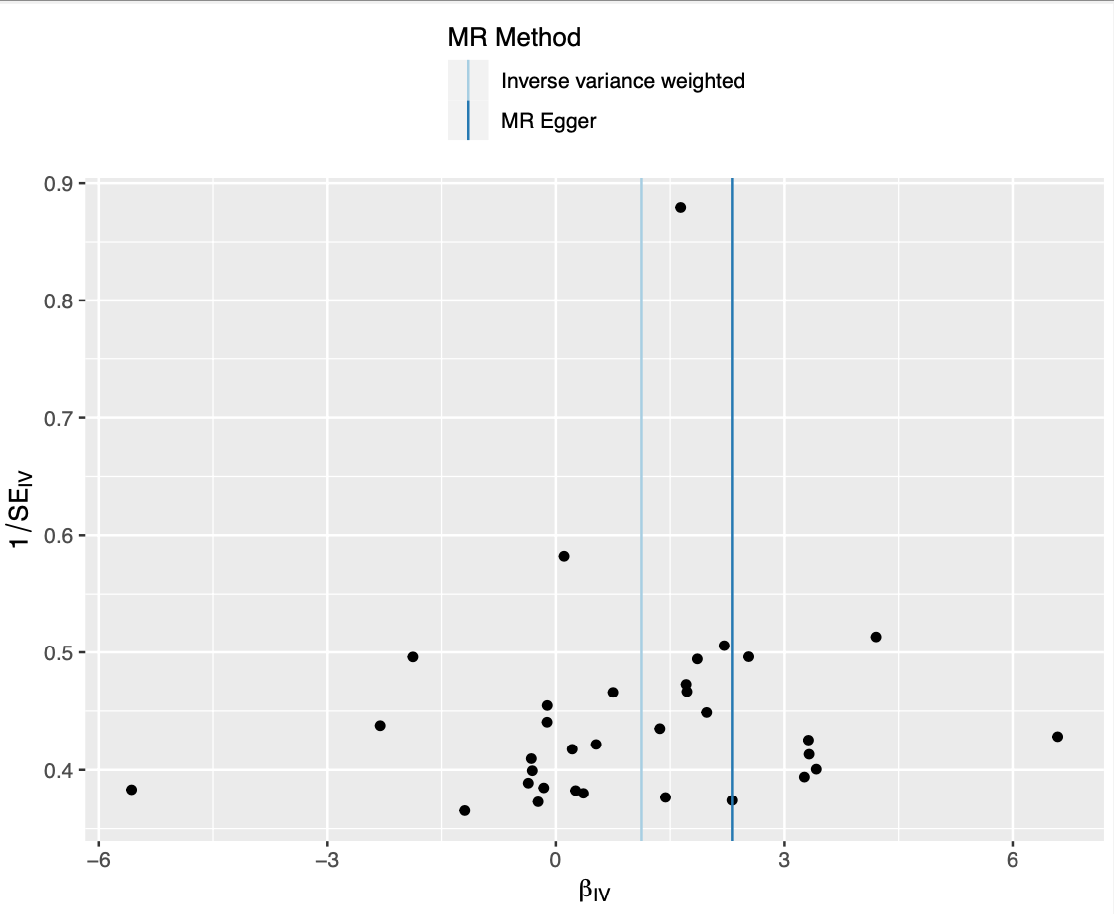


Figure 3. Funnel plots of MR analyses for insomnia on amily Oxalobacteraceae (ID:2966)


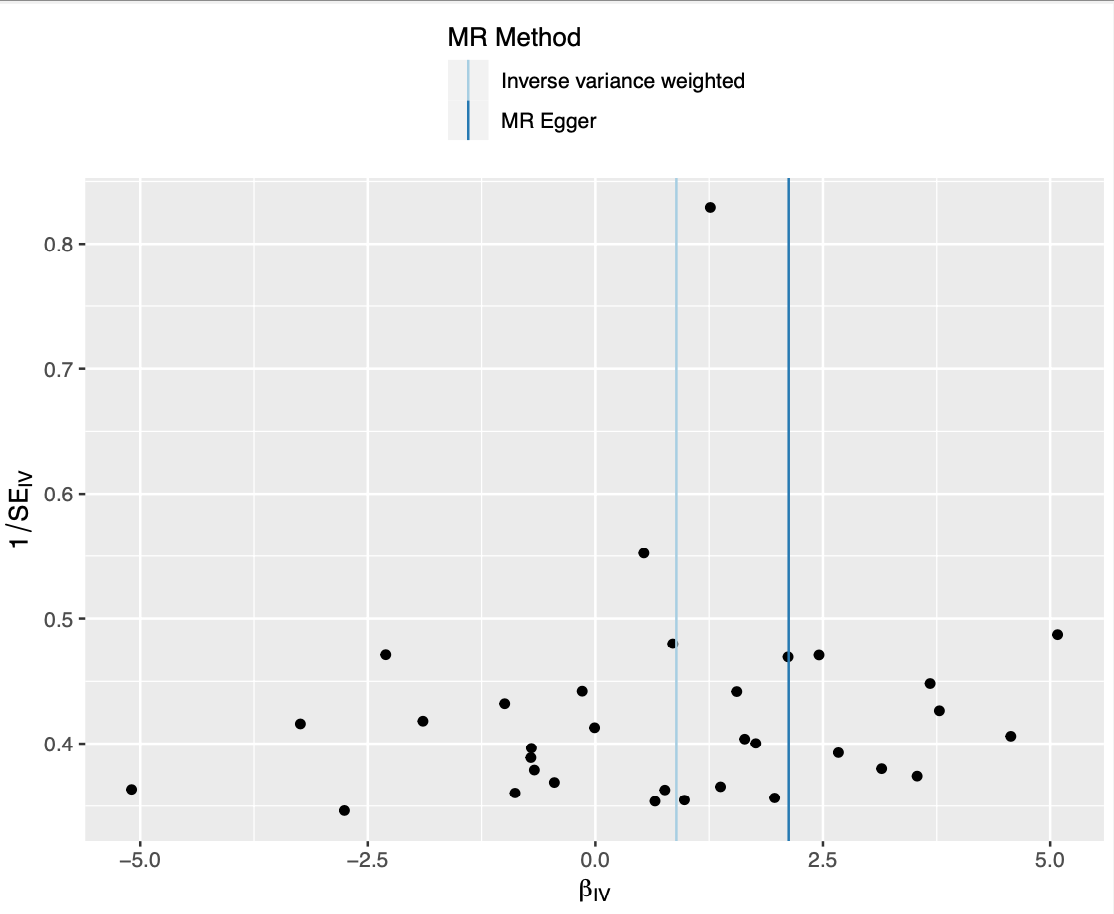


Figure 4 Funnel plots of MR analyses for insomnia on genus *Oxalobacter* (ID:2978)


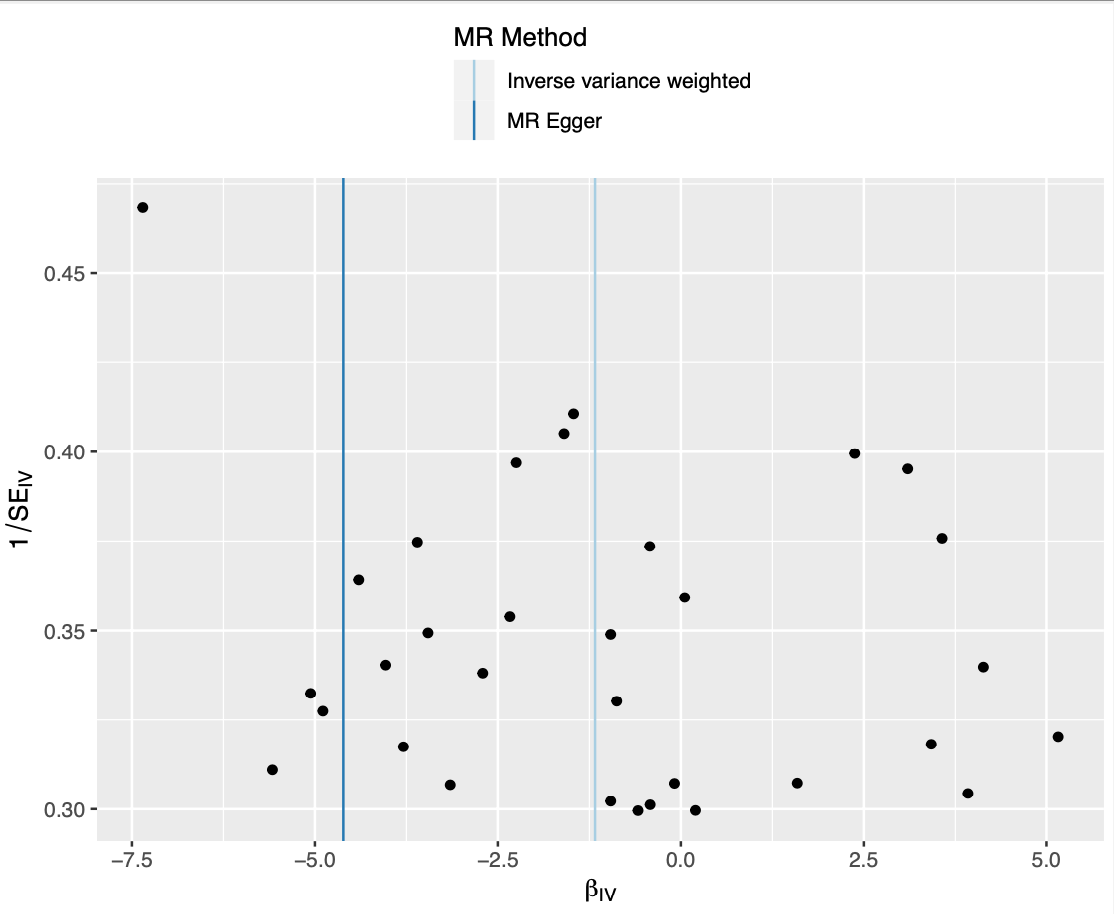


Figure 5. Funnel plots of MR analyses for insomnia on genus *Eubacteriumnodatumgroup* (ID:11297)


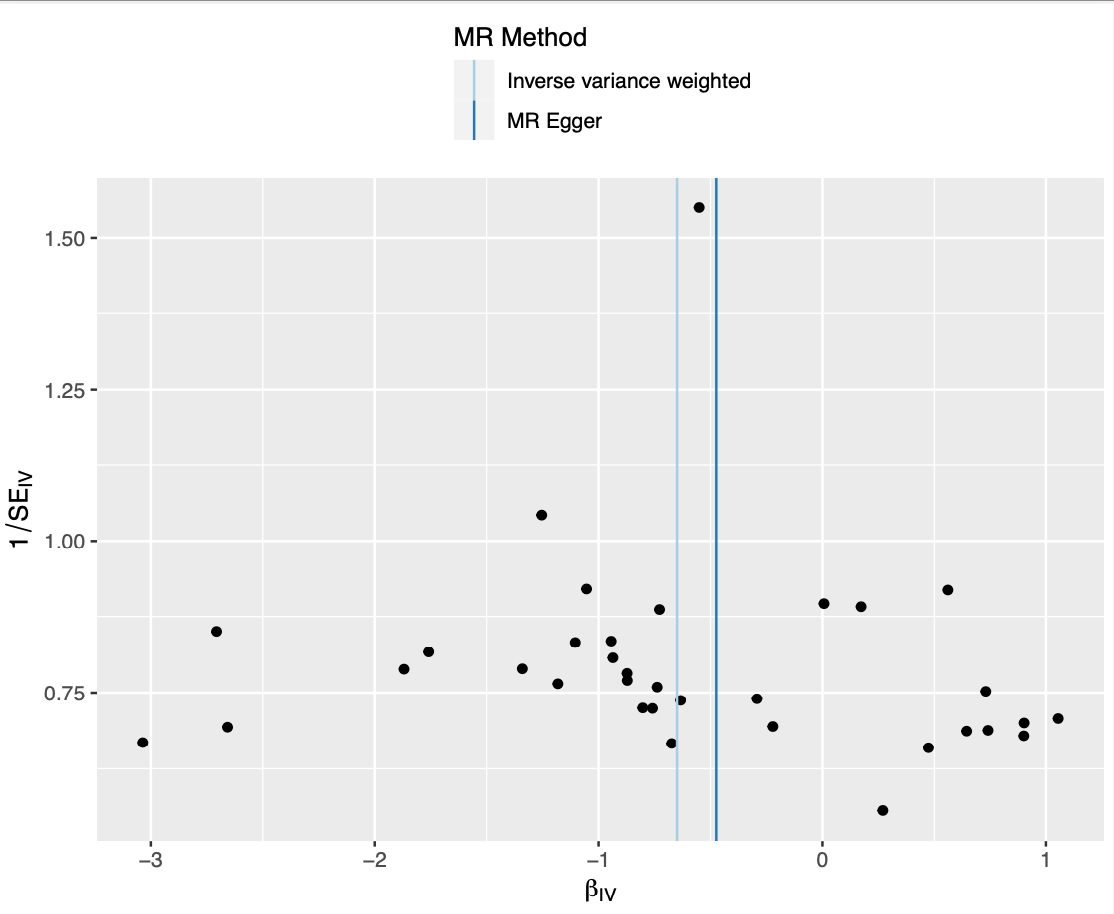


Figure 6. Funnel plots of MR analyses for insomnia on genus *RuminococcaceaeUCG013* (ID:11370)
